# Dietary antigens suppress the proliferation of type 2 innate lymphoid cells by restraining homeostatic IL-25 production

**DOI:** 10.1038/s41598-022-11466-4

**Published:** 2022-05-06

**Authors:** Minji Lee, Hyun-Ja Ko, Sung-Wook Hong, Jungeun Park, Seokjin Ham, Mingyu Kim, Dong-il Kwon, Myeong-seok Lee, Tae-Young Roh, Kwang Soon Kim, You Jeong Lee

**Affiliations:** 1 Department of Life Sciences, Pohang University of Science and Technology (POSTECH), Pohang, Republic of Korea; 2 College of Pharmacy, Research Institute of Pharmaceutical Sciences, Seoul National University, Seoul, Republic of Korea; 3 Present Address: NeoImmuneTech Inc., Pohang, Republic of Korea; 4 Present Address: KoBioLabs Inc., Seoul, Republic of Korea; 5 Department of Microbiology and Immunology, Center for Immunology, University of Minnesota Medical School, Minneapolis, Minnesota USA

**Keywords:** Immunology, Innate immunity, Mucosal immunology

## Abstract

Dietary antigens affect the adaptive immunity of the host by inducing regulatory T cells and IgE-producing B cells. However, their roles in innate immune compartments such as innate lymphoid cells (ILCs) and intestinal epithelial cells (IECs) are unclear. Here, using antigen-free (AF) mice, which are germ-free (GF) mice fed with amino-acid-based diet, we found dietary proteins suppress the development of GATA-3-expressing ILC2s independent of the adaptive immune cells. These cells produce more type 2 cytokines and upregulated proliferation and activation markers such as Ki-67, CD69, and CD25. With this, AF mice had increased expressions of tuft cell-specific transcripts such as *Il25, Il33*, *Dclk1*, *Trpm5*, and *Pou2f3* in IECs. Accordingly, expanded ILC2s upregulated IL-17RB, a receptor of IL-25, and their proliferation was blocked by IL-25 neutralizing or IL-17RB blocking antibodies. These results suggest a new dialogue between dietary antigens, IECs, and ILCs in which dietary antigens suppress ILC2 activation and proliferation by restraining homeostatic IL-25 production, potentially limiting type 2 immunity by food antigens.

## Introduction

There are complex interplays between dietary antigens, commensal microbiome, and host adaptive immunity. For example, dietary antigens induce Nrp1+ regulatory T cells (Tregs) in the small intestine for tolerance induction against food antigens^1^. In addition, they induce IgE-producing B cells in mesenteric lymph nodes of germ-free (GF) mice^2,3^. However, the role of dietary antigens on innate immunity or intestinal epithelial cells (IECs) has not been addressed.

Innate lymphoid cells (ILCs) comprise phenotypically and functionally distinct subtypes—ILC1s, ILC2s, and ILC3s according to expression patterns of surface markers, transcription factors, and cytokine profiles, analogous to Th1, Th2, and Th17 CD4 T cells, respectively^4^. Similar to Th2 CD4 T cells, ILC2s protect hosts against parasite infections^5,6^, but they also promote allergic responses such as food allergy in the gut^7^. The homeostasis of ILCs in the small intestine is regulated by various intestinal contents, including microbiome and chemical metabolites derived from dietary components and micro-organisms. For example, proportions of ILC3s decrease in GF mice, and ILC2s increase in vitamin A and aryl hydrocarbon receptor (AhR) deficient mice^8–10^. So far, however, the role of dietary antigens on the homeostasis of ILCs has not been explored.

Tuft cells have nutrient sensors and expand during helminth infection^6^. They are the source of IL-25 required for activation of ILC2s that produce IL-13, IL-5, and IL-9^6,11–13^. Mechanistically, the end-product of fermentable fibers, succinate produced by *N. brasiliensis* and *Trichomonas* lead to TRPM5-mediated IL-25 production in tuft cells signaling through the succinate receptor, GPR91^14,15^. Tuft cell-derived IL-25 is essential for the expulsion of helminth parasites and stimulates IL-13 production from ILC2s. As a feedback mechanism, IL-13 acts on undifferentiated epithelial progenitors to promote differentiation into tuft cells and goblet cells^16^. Therefore, IL-25 and IL-13 cytokines mediate cross-talk between epithelial and innate immune cells in response to commensals and pathogens. However, the responses of tuft cells on dietary antigens are not defined.

In this study, to address the role of dietary proteins on ILCs, we used antigen-free (AF) mice, which are GF mice fed with an amino-acid supplemented protein-free diet. Interestingly, we found that ILC2s expand and have activated phenotype producing more Th2 type cytokines in the small intestine of AF mice independent of T or B cells. Blocking IL-25 or IL17RB restored the phenotype of ILC2s in AF mice, and as a potential source of IL-25, we found AF mice had increased expression of *Il25, Pou2f3, and Trpm5* in IECs compared to GF or specific-pathogen-free (SPF) mice. These findings suggest that tuft cells produce more IL-25 in the absence of dietary antigens, which subsequently induce the proliferation of ILC2s. However, there was no tuft cell hyperplasia in AF mice and we speculate that the cytokines produced from tuft cells and ILC2s are insufficient to establish a positive feedback loop between them, unlike seen in parasitic infection. The above findings show that dietary antigens suppress the proliferation of ILC2s, which would limit Th2 type immune responses such as food allergies. Collectively, we show a new dialogue between dietary antigens, intestinal epithelial cells, and ILCs regulating type 2 immunity.

## Results

### Dietary proteins suppress the development of ILC2 independent of adaptive immunity

**Figure 1:**
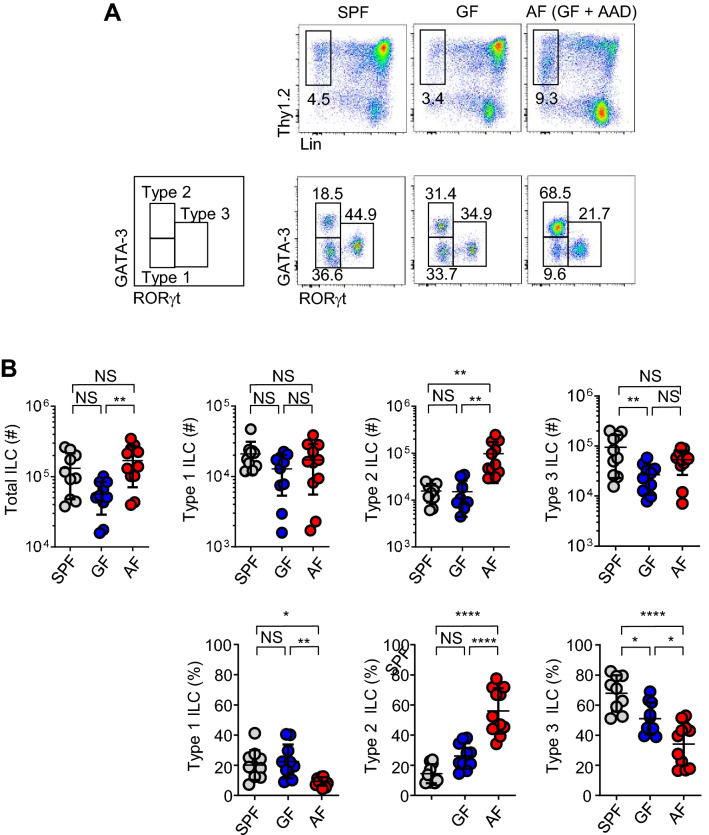
Dietary antigens suppress the development of type 2 ILCs. Single-cell suspensions of the siLP mononuclear cells isolated from young adult SPF, GF, and AF mice were analyzed by flow cytometry. (**A**) Representative dot plots show GATA-3 and RORγt expression on gated Lin^−^ CD45^+^ Thy1.2^+^ cells from indicated mice. (**B**) Graphs show statistical analysis of numbers (upper) and frequencies (lower) of total and each ILC subset from SPF (n=10), GF (n=10), and AF (n=11) mice. Pooled results from three independent experiments are shown. Horizontal bars indicate mean values and error bars show SD. Each dot represents an individual mouse. NS.: not significant, *p<0.05, **p<0.01, ***p<0.001, ****p<0.0001 (one-way ANOVA). siLP, small intestinal lamina propria; Lin, lineage; ILC, innate lymphoid cell; SPF, specific pathogen-free; GF, germ-free; AF, antigen-free; AAD, amino-acid diet; NCD, normal chow diet.

To explore the role of dietary antigens in the homeostasis of ILCs, we used AF mice, which are GF mice fed with an amino-acid-based diet (AAD) as previously described^1^. We used AF mice to exclude any possible effect of intestinal bacteria on host immunity because dietary components influence microbial function and repertoire^17^. Among total mononuclear cells isolated from small intestinal lamina propria (siLP), we gated total ILCs by lineage (CD3, TCRβ, CD4, CD8, B220, CD11b, and CD11c) negative and Thy1.2 positive cells. We further divided them into ILC2 and ILC3 by GATA3 and RORγt expression, respectively, as shown in Fig. 1A. ILC1 was defined as GATA3^−^ RORγt^−^ TBET^+^ cells. Consistent with the previous report^8^, GF mice had decreased numbers of ILC3s and non-significant changes of ILC2s compared to SPF mice (Fig. 1). Interestingly, AF mice had higher numbers and frequencies of ILC2s (GATA-3^+^ RORγt^−^) in siLP. In addition, we found ILCs in AF mice have lower expression levels of Thy1 than SPF and GF mice (Fig. 1A), suggesting they are activated cells. ILC2s are heterogeneous population and previous reports defined ST2+ natural and KLRG1+ Sca-1+ inflammatory ILC2s in the lung^18,19^. We analyzed these markers in the gut found small intestinal ILC2s uniformly expressed KLRG1 and Sca-1 but not ST2, and there were no differences between SPF, GF, and AF mice (Fig. S1A). We speculate that the functional heterogeneity of intestinal ILC2s is different from the lung, which requires further investigation. We also found the number of ILC2 progenitors was not increased in the BM of AF mice, indicating AF diet affects peripheral ILCs (Fig. S1B). ILC3s in AF mice had similar patterns of NKp46 and CCR6 expressions and IL-22 production capacity (Fig. S2), indicating ILC3s in AF mice are not overtly different from those of GF mice. Because AF mice had significantly decreased numbers of CD4 T cells, especially Nrp1 negative Tregs in the small intestine compared to GF mice^1^, we further investigated whether an adaptive immune system affects ILC2 numbers using *Rag1* KO mice (Fig. S3). Similar to WT mice, we found that Rag1 deficient AF mice had increased numbers of ILC2s and decreased frequencies of ILC3 compared to SPF and GF mice. Overall, these results show that dietary proteins suppress the activation and/or proliferation of ILC2s independent of adaptive immunity in the periphery.

### Normal chow diet restores the ILC2 phenotype of AF mice

**Figure 2:**
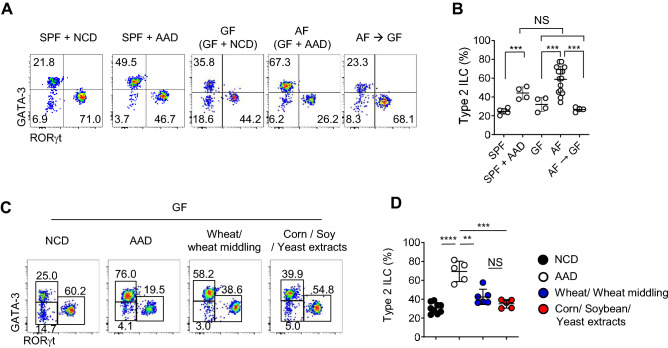
Dietary proteins restore the ILC2 phenotype of AF mice. Single-cell suspensions of the siLP mononuclear cells isolated from mice fed with the indicated diets were analyzed by flow cytometry. (**A** and **B**) SPF or GF mice were fed with the indicated diets. Dietary shift from AAD to NCD in GF mice (AF GF) was at the 6th week and mice were analyzed at the 9^th^ week after birth. Dot plots show ILCs after gating Lin^−^ CD45^+^ Thy1.2^+^ cells (A), and graph shows statistical analysis of frequencies of type 2 ILCs in indicated mice (N = 3~14) (B). (**C** and **D**) GF mice were fed with indicated diet. Dot plots show representative dot plots of ILCs after gating Lin^−^ CD45^+^ Thy1.2^+^ cells (C) and graph shows statistical analysis (N=5~9) (D). Pooled results from two independent experiments are shown. Numbers indicate frequencies of cells in adjacent gates, and each dot represents an individual mouse. Horizontal bars indicate mean values, and error bars show SD. *p<0.05, **p<0.01, ***p<0.001, **** *p*<0.0001. NS, not significant (unpaired two-tailed student *t*-test). siLP, small intestinal lamina propria; ILC, innate lymphoid cell; SPF, specific pathogen-free; GF, germ-free; AF, antigen-free; NCD, normal chow diet; AAD, amino-acid diet.

Next, we tested whether dietary shift can restore the phenotype of ILCs in AF mice by feeding six-week-old AF mice with normal chow diet (NCD) for an additional three weeks (Fig. 2A). Upon the dietary transition, the frequencies of ILC2s were restored in the small intestine (Fig. 2A,B). We used AF mice to completely rule out the possible role of the commensal microbiome because AAD alters their repertoire^1^. However, SPF mice fed with AAD also showed increased frequencies of ILC2s in the small intestine suggesting an altered microbiome has minimal impacts on ILCs. Tuft cells provide IL-25 and regulate the expansion of ILC2s^6^, and they have taste receptors, including TAS1R3, which senses umami by glutamate^20^. Therefore, we further tested whether free amino acids in AAD directly influence the tuft cell—ILC2 axis by adding glutamate or whole AAD in drinking water (Fig. S4). If AAD directly affects the tuft cell—ILC2 axis, this mouse should have increased ILC2s in the gut, similar to AF mice. However, these mice had no alteration in numbers and frequencies of ILC2s, indicating the free amino acids do not directly alter the phenotype of ILCs. Next, we asked whether specific ingredients of NCD suppress the expansion of ILC2s in SPF mice. NCD includes six major dietary components: ground corn, corn gluten meal, wheat, wheat middling, soybean meal, and yeast extracts. Previous reports showed that dietary gluten induces IL-4 producing T follicular helper 2 (T_FH_2) cells and IgE producing B cells^3^. Therefore, we divided NCD into two groups, wheat/wheat middling that include gluten and corn/soybean/yeast extracts that do not contain gluten (Fig. 2C,D). As a result, we found GF mice fed with both groups had significantly decreased frequencies of ILC2s compared to AF mice. Therefore the normalization of ILC2s in AF to GF shift (Fig. 2A,B) is mediated by the six major protein ingredients, not by minor contaminants of NCD diet, and gluten does not specifically induce the expansion of ILC2s. Collectively, these findings show that various sources of dietary proteins suppress the proliferation of ILC2s in the small intestine.

### ILC2s of AF mice have activated and proliferating phenotypes

**Figure 3:**
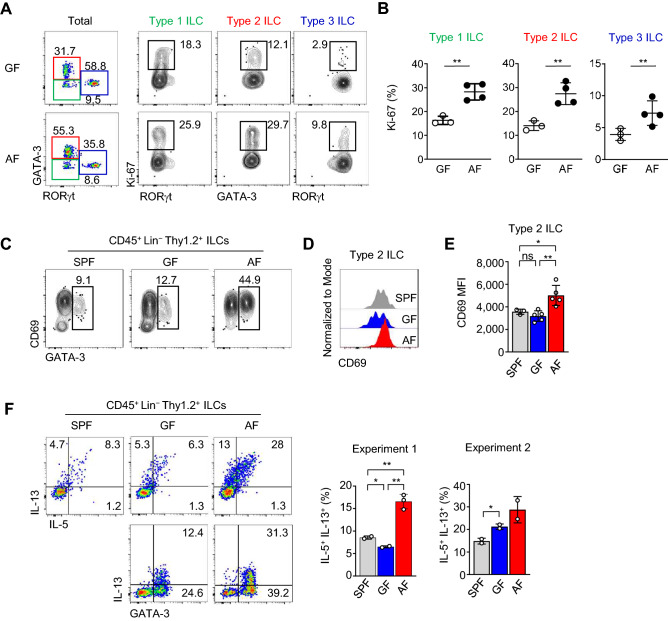
ILC2s of AF mice have activated and proliferating phenotypes. Single-cell suspensions of the siLP mononuclear cells isolated from indicated mice were analyzed by flow cytometry. (**A**–**B**) Dot plots show the expressions of Ki-67 in each ILC subset in GF and AF mice (A). Graphs show statistical analysis of Ki-67 expression in ILC populations in the lamina propria of the gut from GF or AF mice. (**C**) Contour plots show CD69 expressions in GATA-3+ type 2 ILCs of indicated mice. (**D**–**E**) The histogram shows CD69 expressions (D), and the graph shows statistical analysis of CD69 MFI (N=4~5) in the indicated mice (E). (**F**) Dot plots show intracellular effector cytokines (IL-13, and IL-5) in gated Thy1.2+ Lin– total ILCs from the siLP of indicated mice after 4 hr stimulation with PMA and ionomycin in the presence of Brefeldin A and Monensin. Representative data from three independent experiments are shown. Numbers indicate frequencies of cells in adjacent gates, and each dot represents an individual mouse. Horizontal bars indicate mean values, and error bars show SD. *p<0.05, **p<0.01. NS, not significant (unpaired two-tailed student t-test). siLP, small intestinal lamina propria; ILC, innate lymphoid cell; SPF, specific pathogen-free; GF, germ-free; AF, antigen-free.

Next, we analyzed the phenotypes of expanded ILC2s in the small intestine of AF mice (Fig. 3). Ki-67, a nuclear marker of proliferating cells, was highly expressed in ILC2s (Fig. 3A,B) with upregulation of early activation marker CD69 (Fig. 3C–E), indicating that AAD promotes rapid turnover and activation of ILC2s. Indeed, expanded ILC2s secreted more type 2 cytokines such as IL-5 and IL-13 than SPF and GF mice (Fig. 3F). Therefore, the expansion of ILC2s in AF mice accompanied their proliferation, upregulation of activation markers, and enhanced cytokine secretion.

### Small intestinal epithelial cells of AF mice upregulate tuft cell-specific markers

**Figure 4:**
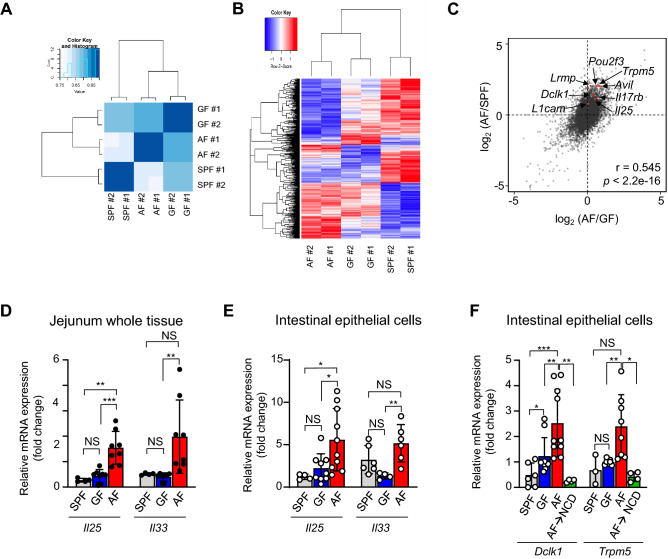
Small intestinal epithelial cells of AF mice upregulate tuft cell-specific markers. (**A**–**C**) Small intestinal epithelial cells (Live CD45^−^ EpCAM^+^) were sorted from SPF, GF, and AF mice as duplicate, and RNA sequencing was performed. Heat maps show the correlation (A) and the unsupervised hierarchical clustering of gene expression levels (B) of the 811 DEGs in each group. (C) Dot plot shows the fold change of gene expression levels. The X-axis represents the log2 fold-change of DEGs found by comparative AF and GF groups. Y-axis represents log2 fold change of DEGs found by comparative AF and SPF groups. Each dot represents an individual gene. (**D**–**F**) Real-time qPCR analysis was performed for indicated genes using whole tissue lysate of the jejunum (D) and sorted small intestinal epithelial cells (Live CD45^−^ EpCAM^+^) (E and F). C*t* values were normalized to the expression levels of 18s ribosomal RNA. *p<0.05, **p<0.01. ***p<0.001 NS, not significant (unpaired two-tailed student *t*-test). Error bars indicate SD. SPF, specific pathogen-free; GF, germ-free; AF, antigen-free.

Intestinal content directly affects the transcriptional nature of IECs, as shown in GF mice that have low levels of *Reg3g* expressions in them^21^. Based on this, we speculated that the absence of dietary antigens would also influence the IECs, contributing to the phenotype of ILC2s. To address this issue, we isolated IECs from SPF, GF, and AF mice from two biological replicates and performed RNAseq analysis (Fig. 4). The gene expression pattern of the two replicates was well correlated in each SPF, GF, and AF group (Fig. 4A), and we found a total of 811 differentially expressed genes (DEGs) (Fig. 4B and Table S1). Interestingly, genes that are upregulated in AF mice compared to SPF mice were also upregulated compared to those of GF mice (Fig. 4C), indicating that dietary protein deficiency induces similar transcriptional changes of IECs both in SPF and GF conditions. Using this plot, we listed upregulated genes in AF mice compared to both SPF and GF mice (Fig. 4C). These genes included *Pou2f3, Trpm5, Il25, Il17rb,* and *Dclk1,* which are unique markers of tuft cells^6,14,22^. To validate this result, we used whole jejunum or isolated IECs from SPF, GF, and AF mice and performed real-time qPCR analysis (Fig. 4D–F). Consistent with RNAseq experiments, we confirmed that IECs from AF mice had higher expression levels of *Il25, Dclk1,* and *Trpm5* than SPF and GF mice. In addition, we found *Il33* was also significantly upregulated in IECs of AF mice compared to GF mice. These results indicate that, in the absence of dietary antigens, IECs produce alarmins such as IL-25 and IL-33^6^.

### Expansion of ILC2s in AF mice is dependent on IL-25

**Figure 5:**
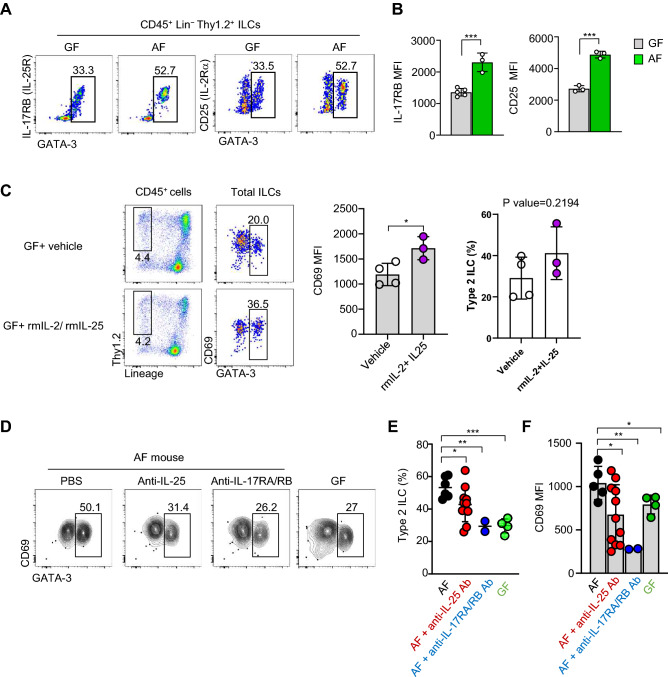
Expansion of ILC2s in AF mice is dependent on IL-25. Single-cell suspensions of the siLP mononuclear cells isolated from indicated mice were analyzed by flow cytometry. (**A** and **B**) Dot plots show IL-17RB and CD25 expressions in CD45^+^ Lin^−^ Thy1.2^+^ cells in the indicated mice (A). Graphs show statistical analysis (N=3~5) (B). Data are representative of three independent experiments. (**C**) GF mice were administered with either PBS (vehicle) or recombinant mouse (rm) IL-25 plus IL-2 daily for three consecutive days. Dot plots show CD69, and Ki-67 expressions in ILC2s, and the graph shows statistical analysis of CD69 expressions. (**D**–**F**) Contour plots show frequencies of ILC2s and their CD69 expressions in AF mice administrated with 250µg anti-IL-25 or anti-IL-17RA/RB-blocking antibodies for five consecutive days (D). Graphs show statistical analysis of the frequencies of ILC2s (E) and CD69 MFIs (F). Results were pooled from 3 independent experiments (N=2~11). *p<0.05, **p<0.01. ***p<0.001. NS, not significant (unpaired two-tailed student t-test). GF, germ-free; AF, antigen free; Lin, Lineage; MFI, mean fluorescence intensity.

Based on the above results, we hypothesized that increased IL-25 and IL-33 from IECs in AF mice would drive the proliferation of ILC2s in the gut. To test this possibility, we first checked the expression of IL-17RB (IL-25 receptor), and CD25 (IL-2 receptor α) upregulated by IL-33^23^. As expected, there were upregulations of IL-17RB and CD25 in ILC2s of AF mice compared to those of GF mice (Fig. 5A,B). Next, we tested whether ILC2s upregulate CD69 in response to exogenous IL-25. To address this issue, we injected IL-25 to GF mice with IL-2 as the previous report showed IL-25 alone is not sufficient for ILC2 stimulation in a short-tem period^24,25^. Three days after three consecutive injections, we found that this cytokine-stimulated ILC2s upregulated CD69 (Fig. 5C dot plots and left graph). However, the number of ILC2s was not significantly increased in this mouse (Fig. 5C, right graph). It is possible that analysis at a later time would induce ILC2 proliferation, as the previous report showed that activation of tuft cell—ILC2 axis by exogenous IL-25 requires chronic injection over four weeks^26^. Next, we tested the effect of blocking antibodies against IL-25 and its receptor IL-17RB. Intraperitoneal (i.p.) injections of anti-IL-25 (IL-17E) or anti-IL-17RA/RB-blocking antibodies for five consecutive days in AF mice decreased the numbers of ILC2s to a comparable level of GF mice with downregulation of CD69 (Fig. 5D–F). Collectively, these results indicate that IL-25 increases the numbers of ILC2s, and the expansion of ILC2s in AF mice is restored by blocking IL-25 signaling.

Because expanded ILC2s secrete IL-13 (Fig. 3F), they might induce the proliferation of tuft cells through a feedback mechanism as previously shown^6,14^. However, we found no difference in the frequency of DCLK1 positive cells in the small intestine between GF and AF mice (Fig. S5). Consistent with this, genes associated with goblet cells such as *Gfi1, Muc2, Spdef, Tff3*^27^ were not increased in IECs of AF mice (Table S1), and there no overt goblet cell hyperplasia in AF mice (Fig. S5B),. Therefore, we speculate that expanded ILC2s in AAD diet mice do not produce sufficient IL-13 required for the proliferation of tuft cells as a feedback mechanism.

## Discussion

In this study, we showed that ILC2s expand in GF or SPF mice fed with AAD. They upregulated CD69, CD25, Ki-67, and IL17RB and produced IL-5 and IL-13, indicating they actively proliferate and secrete cytokines. Mechanistically, we found increased production of IL-25 from IECs, and blocking antibodies against IL-17 or IL-25 receptor (IL-17RB) restored the phenotype of ILC2s in AF mice. Although tuft cell-specific transcripts were increased in IECs, there was no tuft cell hyperplasia in AF mice. We speculate that IL-13 produced from expanded ILC2s was below the threshold. In the future, tuft cell-specific IL-25 deletion would provide definite evidence that tuft cells are the source of IL-25 inducing the proliferation of ILC2s in AF mice. Regardless of the origin of IL-25, however, our study suggests that the absence of dietary protein in the gut expands ILC2s, which would make our body more responsive to type 2 immunity.

We specifically designed AF mice to study the role of dietary antigens without causing nutritional imbalances. In this study, we used a liquid form of AF diet supplemented with essential amino acids, fatty acids, and carbohydrates made in our facility as previously described^1^. When AAD fed with NCD, there was no influence on the numbers of ILCs, suggesting AAD does not directly influence activation and/or proliferation of ILC2s (Fig. S3). Therefore expanded ILC2s in AF mice is more likely to be due to the absence of dietary proteins. Consistent with this idea, mice fed with major ingredients of dietary protein sources in NCD restored the phenotype of ILC2s in AF mice (Fig. 2). These results exclude the possible direct effects of free amino acids or indirect impacts of minor ingredients of AAD in the expansion of ILC2s in AF mice.

A previous study showed ILC2s expand and ILC3s decrease in vitamin A deficient (VAD) mice by enhanced expression of IL-7 receptors in ILC2 progenitors^9^. As a result, these mice exhibited augmented type 2 immunity with goblet cell hyperplasia resulting in more resistance to parasitic infections. However, at the same time, they become more vulnerable to bacterial infections due to the loss of ILC3s. In this mouse, tuft cells were not analyzed, and they did not test ILC2 extrinsic effects that might determine the phenotype of VAD as it causes systemic deficiency of vitamin A. In contrast, we used AF diets that supplied all necessary nutritional ingredients, ruling out systemic side-effects caused by nutritional imbalance. Overall, these results show the dietary components affect ILC homeostasis.

Tuft cells act as a chemosensory receptor and orchestrate type 2 immunity against parasitic infections^6,14,22^. Small intestinal DCLK1^+^ Trpm5^+^ tuft cells were markedly accumulated and secreted IL-25 during colonization of helminth parasites, such as *Nippostrongylus brasiliensis* or *Trichinella spiralis*. Because these worms compete against the host for nutrients, the AF diet may mimic the condition of parasite infection. In this perspective, accumulated ILC2s in AF mice might decrease worm burden after parasite infection. In the future study, it would be interesting to check whether the AF diet promotes anti-parasite immunity by expanding ILC2s.

## Materials and methods

### Mice

GF C57BL/6 mice were previously described^1,28^, and SPF C57BL/6 mice were purchased from Jackson Laboratory. For the generation of AF mice, GF B6 mice were fed either with an antigen-free diet as previously described^1^. This research was approved by the Institutional Animal Care and Use Committees (IACUC) of the Pohang University of Science and Technology (POSTECH) in accordance with ARRIVE guidelines (https://arriveguidelines.org). Mouse care and experimental procedures were performed in accordance with all institutional guidelines for the ethical use of non-human animals in research and protocols from IACUC of the Pohang University of Science and Technology.

### Antibodies and flow cytometry

Single-cell suspensions of small intestinal mononuclear cells were washed with PBS and stained with Ghost viability dye to distinguish live and dead cells. For surface staining, cells were incubated with the following antibodies from eBioscience, Biolegend, Tonbo, and BD Biosciences: anti-CD16/32(93), Anti-CD3ε (145-2C11), anti-CD4 (RM4-5), anti-CD8α (53-6.7), anti-CD11c (N418), anti-B220 (RA-3-6B2), anti-TCRβ (H57-597), anti-CD45 (30-F11), anti-Thy1.2 (53-2.1), anti-CD69 (H1.2F3), anti-IL-25R (MUNC33) dissolved in flow cytometry buffer (PBS containing 1% FBS and 0.025% sodium azide). A dump channel (CD3, TCRβ, CD4, CD8, B220, CD11b, and CD11c) was used to exclude undesired mature cells that express lineage markers. For intracellular staining of RORγt (B2D), GATA-3 (TWAJ), IFN-γ (XMG1.2), IL-5 (TRFK5) IL-13 (eBio13A), IL-22 (Poly5164) surface stained cells were fixed and permeabilized with a Foxp3 staining kit (eBioscience) following the manufacturer’s instruction. For intracellular cytokine staining, isolated cell suspensions were culture for 4 hours in RPMI-1640 medium containing 10% FBS, 100 units/ml penicillin, 100 mg/ml streptomycin, and 55 mM β-mercaptoethanol in the absence or presence of phorbol 12-myristate 13-acetate (PMA), ionomycin and protein transport inhibitors (Brefeldin A and Monensin, eBioscience). To induce IL-22 production, the cells were stimulated with 40 ng/ml recombinant IL-1β and 20 ng/ml IL-23. Cells were analyzed using LSRII Fortessa (BD Biosciences) and data were processed with FlowJo software 10.4.1 (Tree Star Inc.).

### Tissue preparation

Mononuclear cells from the small intestine was prepared as previously described^29^. Briefly, the small intestine was removed and put in a petri dish containing ice-cold phosphate-buffered saline (PBS). Fat and Peyer’s patches were removed, and the intestine was opened longitudinally. The intestine was cut into small pieces and incubated (37°C, 30 min) in lamina propria (LP) buffer (DPBS with 3% fetal bovine serum (vol/vol), 10mM EDTA, 20mM HEPES, 1% penicillin/ streptomycin (wt/vol) and 1mM sodium pyruvate) with agitation to remove an intestinal epithelial layer. To isolate intestinal epithelial cells, the released cells were subjected to density gradient centrifugation (RT, 805× g for 20 min) with 25% and 40% Percoll (GE Healthcare). The resulting single-cell suspension at the interphase was collected and placed in Dulbecco’s Modified Eagle’s Medium (DMEM, Welgene) containing 2% fetal bovine serum. For lamina propria preparations, the retained tissue pieces were extensively washed with PBS. Tissue pieces were then collected, diced, and incubated (37°C, 1 hr) with shaking in digestion buffer (RPMI-1640 with 3% fetal bovine serum, 20mM HEPES, 1mM sodium pyruvate, 1mM non-essential amino acids, 1% penicillin/streptomycin (wt/vol), 400 Mandl units/ml Collagenase D (Roche Diagnostics, Risch-Rotkreuz, Switzerland) and 100µg/ml DNase (Biosesang). Digested suspensions were then centrifuged, and the resulting cell pellet was resuspended in 5ml 70% Percoll followed by overlay onto 40% Percoll to enrich intestinal lamina propria cells. In a final step, the 40/70 interphase of the Percoll gradient was harvested, washed, and stained for flow cytometry analysis.

### Real-time qPCR

RNA was extracted and analyzed as previously described^30^. Briefly, RNAs were extracted from the whole jejunum or isolated IECs using Trizol (Invitrogen). cDNA was generated by using a Quantitect reverse transcription kit for cDNA synthesis kit (Qiagen). The synthesized cDNA was stored at -80°C before subsequent analysis. Pre-designed Taqman™ (ThermoFisher) primers were used to quantify the expression of mouse Il25 (Mm00499822_m1), Il33 (Mm00505403_m1), Tslp (Mm01157588_m1), Dclk1 (Mm00444950_m1) and Trpm5 (Mm01129032_m1) using real-time quantitative PCR with Taqman master mix (EagleTaq universal MMX w/ROX, Roche) and ViiA™ 7 Real-time PCR system (Applied Biosystems). The relative expression of the gene of interest was normalized relative to 18s ribosomal mRNA levels and calculated using the 2^-^^ΔΔCt^ method.

### RNA-Seq analysis

Epicam positive 10^6^  small intestinal epithelial cells were sorted with FACS Aria from B6 SPF, GF, and AF mice. An RNeasy kit (Qiagen, Redwood City, CA) was used to isolate RNA obtained from each sample, and RNA sequencing was done at the Macrogen (http://www.macrogen.com) using HiSEquation 2000 (Illumina). The sequencing quality was examined by FastQC^31^. To map the reads to the mouse reference genome and transcriptome (GRCm38_ensGene_89), a STAR-2.5.2b1 aligner was used. The assignment of mapped reads corresponding to genes and the quantification of isoform abundances was performed by RSEM-1.3.0^32^. Using the Ensembl genes annotation database, the transcripts were annotated and normalized by count per million (CPM) and transcript per million (TPM)^33^. Differential expression analysis between different conditions was performed with Bioconductor packages, edgeR-3.14.05 and limma-3.28.20^34^. Only the genes with at least 1 CPM, evaluated at least in 3 samples, were used for further analysis after excluding low count transcripts. Differentially expressed genes were defined as those with a fold change greater than 2 and a p-value less than 0.05. When comparing 3 conditions simultaneously, genes were tested by the one-way analysis of variance (ANOVA) with a p-value less than 0.05. The enrichment analysis of KEGG pathways related to differentially expressed genes was performed using the Enrichr (http://amp.pharm.mssm.edu/Enrichr/)^35^. The heatmap generated by differential expressed genes of pairwise comparisons with ggplot2 function. All plots were drawn in R program (ver3.4.0). Data were deposited with accession number PRJNA487175.

### Cytokine administration

GF C57BL/6 mice were injected intraperitoneally (i.p.) with 1 μg mouse recombinant (rm) IL-25 (R&D systems, 1399-IL-025) in combination with 0.5 μg mouse rmIL-2 (PROSPEC, CYT-370) for three consecutive days and analyzed three days after last injection. The control animals were treated with the vehicle alone.

### IL-25 neutralization

Anti-IL-25 (IL-17E) neutralizing antibody and anti-IL-17RA/RB blocking antibody were a gift of AMGEN Inc. AF mice were treated intraperitoneal injection with 250μg of an anti-IL-25 neutralizing monoclonal Ab or anti-IL-17RA/RB for consecutive six days. Small intestines were harvested on day 7 for flow cytometry analysis.

### Immunofluorescence

Swiss-rolled small intestine was stained as previously indicated^36^. Briefly, tissue was fixed in 4% paraformaldehyde (PFA) for 1h, washed with PBS and left in 30% sucrose overnight before embedding in OCT. Slides were stained with indicated antibodies and DAPI before mounting. Images were obtained using Leica DM6B.

### Statistical analyses

Data were analyzed by the Student t-test using GraphPad Prism software (San Diego, CA). P values less than 0.05 were considered to be statistically significant.

## Supplementary Information

Supplementary Information 1.

Supplementary Information 2.

Supplementary Information 3.
