# Supplementary material for: Dietary antigens suppress the proliferation of type 2 innate lymphoid cells by restraining homeostatic IL-25 production
Source: Sci Rep. 2022 May 6;12:7443. doi: 10.1038/s41598-022-11466-4 (PMC9076687; doi:10.1038/s41598-022-11466-4)
Supplement: Supplementary file 1 — Supplementary Information 1. [file 41598_2022_11466_MOESM1_ESM.pdf]

Supplementary Figures

Dietary antigens suppress the proliferation of type 2 innate lymphoid cells by restraining homeostatic IL-25 production

Minji Lee, Hyun-Ja Ko, Sung-Wook Hong, Jungeun Park, Seokjin Ham, Mingyu Kim, Dong-il Kwon, Tae-Young Roh, Kwang Soon Kim, You Jeong Lee

Figure S1. Profiles of ILCs in AF mice

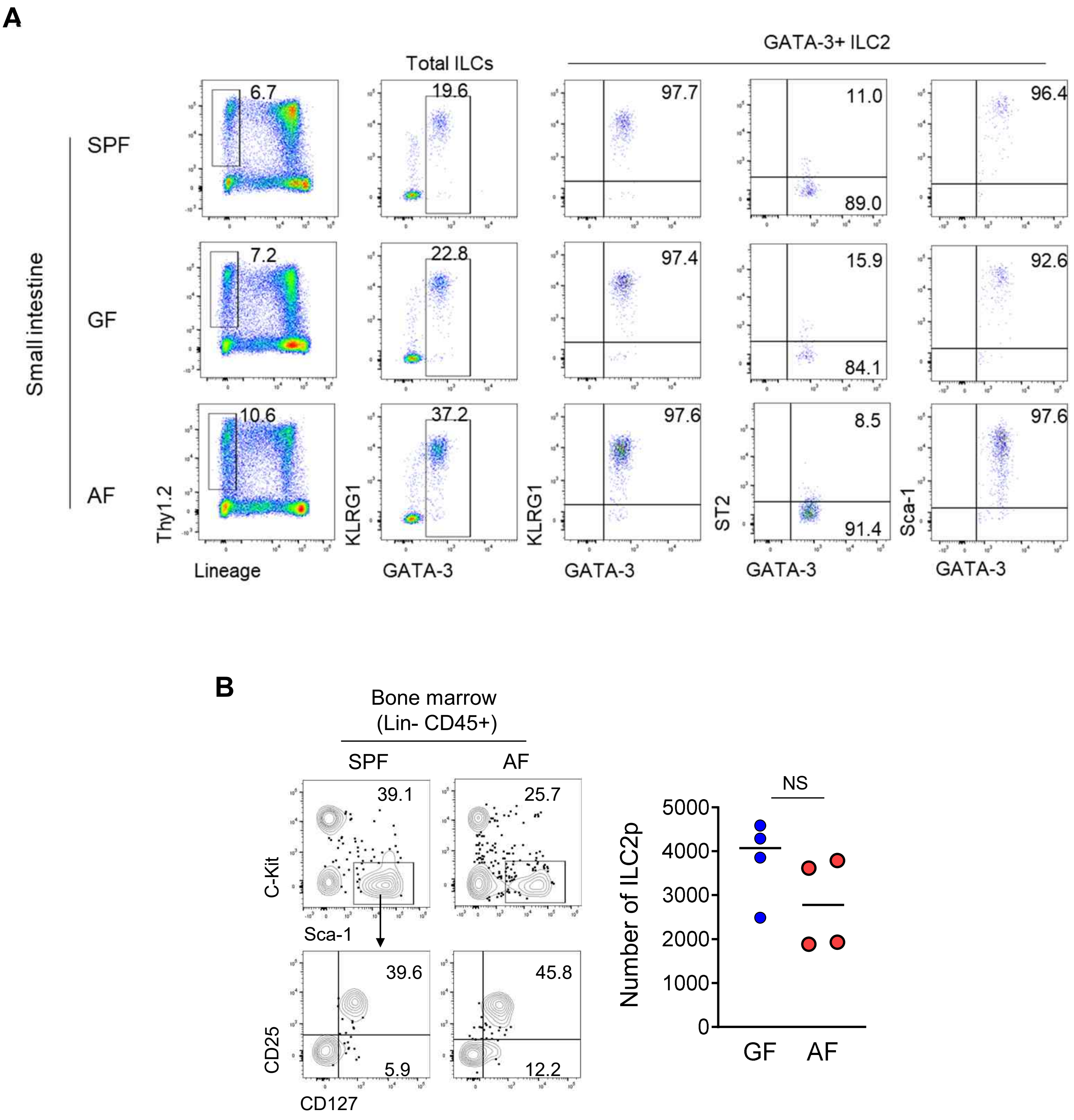

(A) Representative FACS plots show expression of indicated markers in siLP ILC2s of SPF, GF and AF mice. (B) Representative facs plots show ILC progenitors in the bone marrow. Numbers indicate frequencies of cells in adjacent quadrants. Graph shows statistical analysis. NS, non-significant ($p>0.05$). ILC2p, innate lymphoid cell 2 progenitor.

Figure S2. ILC3 of AF mice have similar phenotypes with those of GF mice.

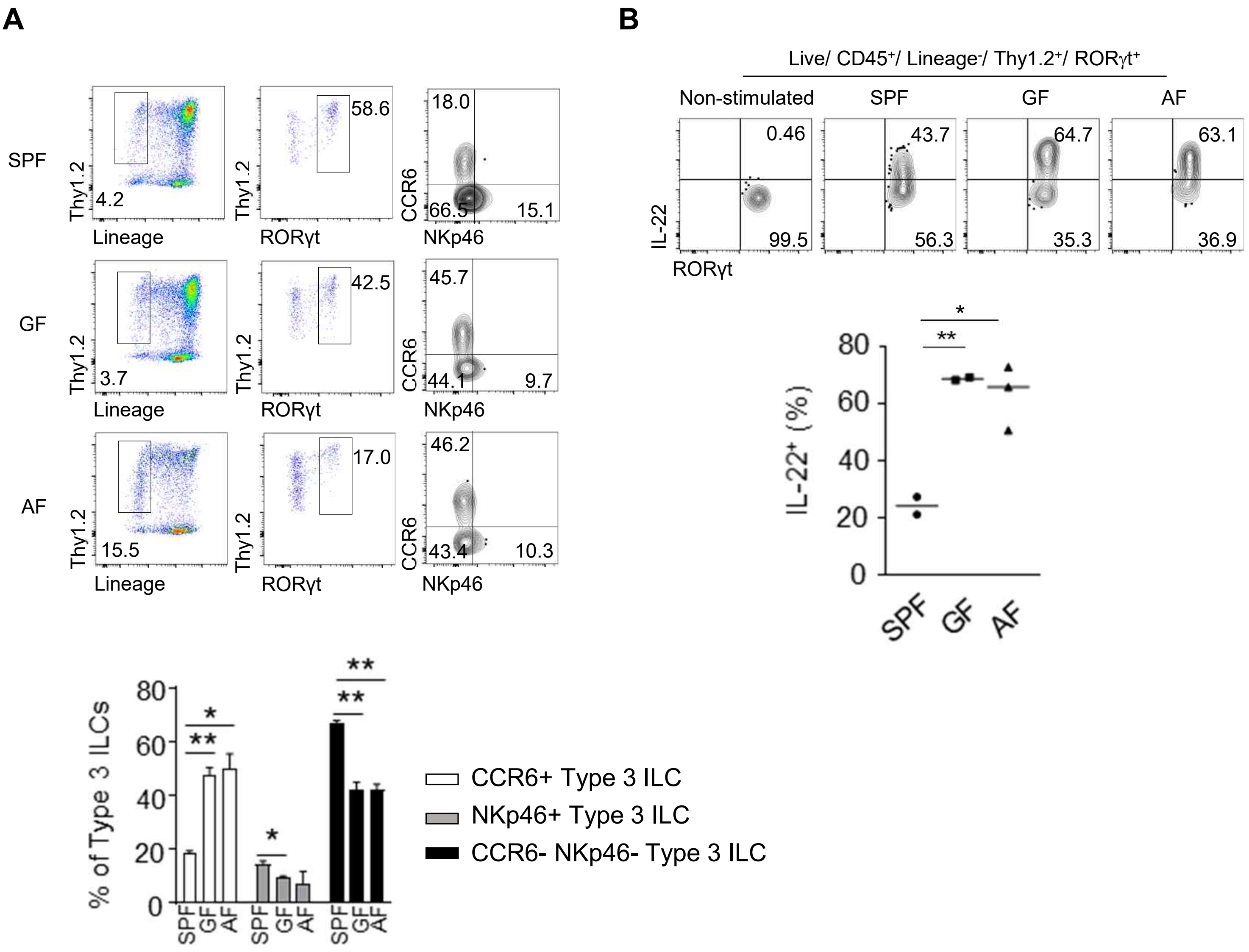

Flow cytometric analysis of ILCs in the siLP of young adult SPF, GF and AF mice. (A) Representative FACS plots to depict heterogenous type 3 ILCs depending on the expression of CCR6 and NKp46. Graph shows statistical analysis (N=3). (B) Shown are representative plots of IL-22 in $CD45^+$ $Lineage^-$ $Thy1.2^+$ $ROR\gamma t^+$ type 3 ILCs from the siLP of indicated mice after 4 hr stimulation with recombinant IL-1β and IL-23 in the presence of Golgi blockers brefeldin A and monensin. Graph shows statistical analysis (N=2-3).

Figure S3. Accumulation of ILC2 of AF mice in siLP is independent of adaptive immune cells.

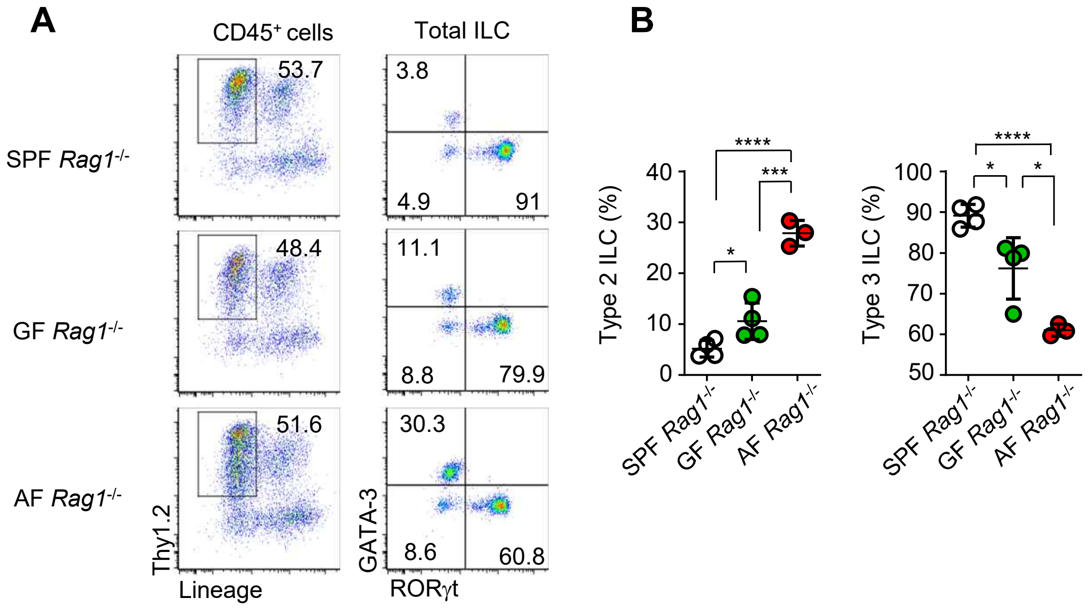

Single-cell suspensions of the siLP mononuclear cells isolated from indicated mice were analyzed by flow cytometry. (A) Dot plots show the distribution of ILCs in indicated mice. (B) Graphs show the statistical analysis of frequencies of type2 and type3 ILCs among total ILCs (N=3~4). Results from two independent experiments are shown. Each dot represents an individual mouse. Horizontal bars indicate mean values and error bars show SD. **p*<0.05, ***p*<0.01. ****p*<0.001 NS, not significant (unpaired two-tailed student *t*-test). siLP, small intestinal lamina propria; ILC, innate lymphoid cell; SPF, specific pathogen-free; GF, germ-free; AF, antigen-free.

Figure S4. Excessive amino acids in the NCD diet do not induce the expansion of ILC2s.

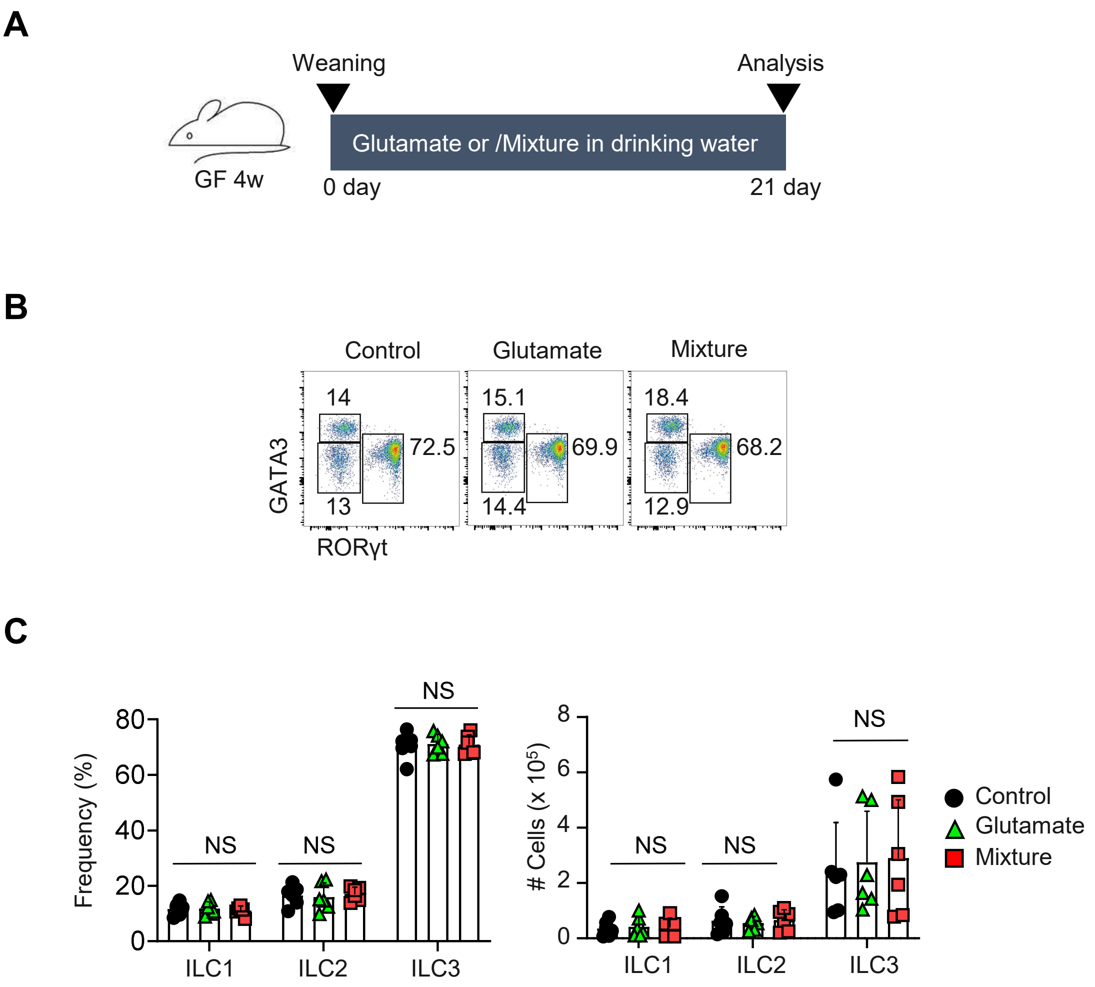

GF mice were fed with glutamate or amino-acid mixture used in AF mice in drinking water for three weeks after weaning. (A) The schematic figure shows the experimental plan. (B) Dot plots show representative data analyzing ILCs gated on CD45.2+ Lin- Thy1.2+ cells in indicated mice. (C) Graphs show frequencies and numbers of each ILC subset in indicated mice (N=6 for each group). Pooled results from 3 independent experiments. NS, not significant (one-way ANOVA).

Figure S5. Tuft cells do not proliferate in AF mice

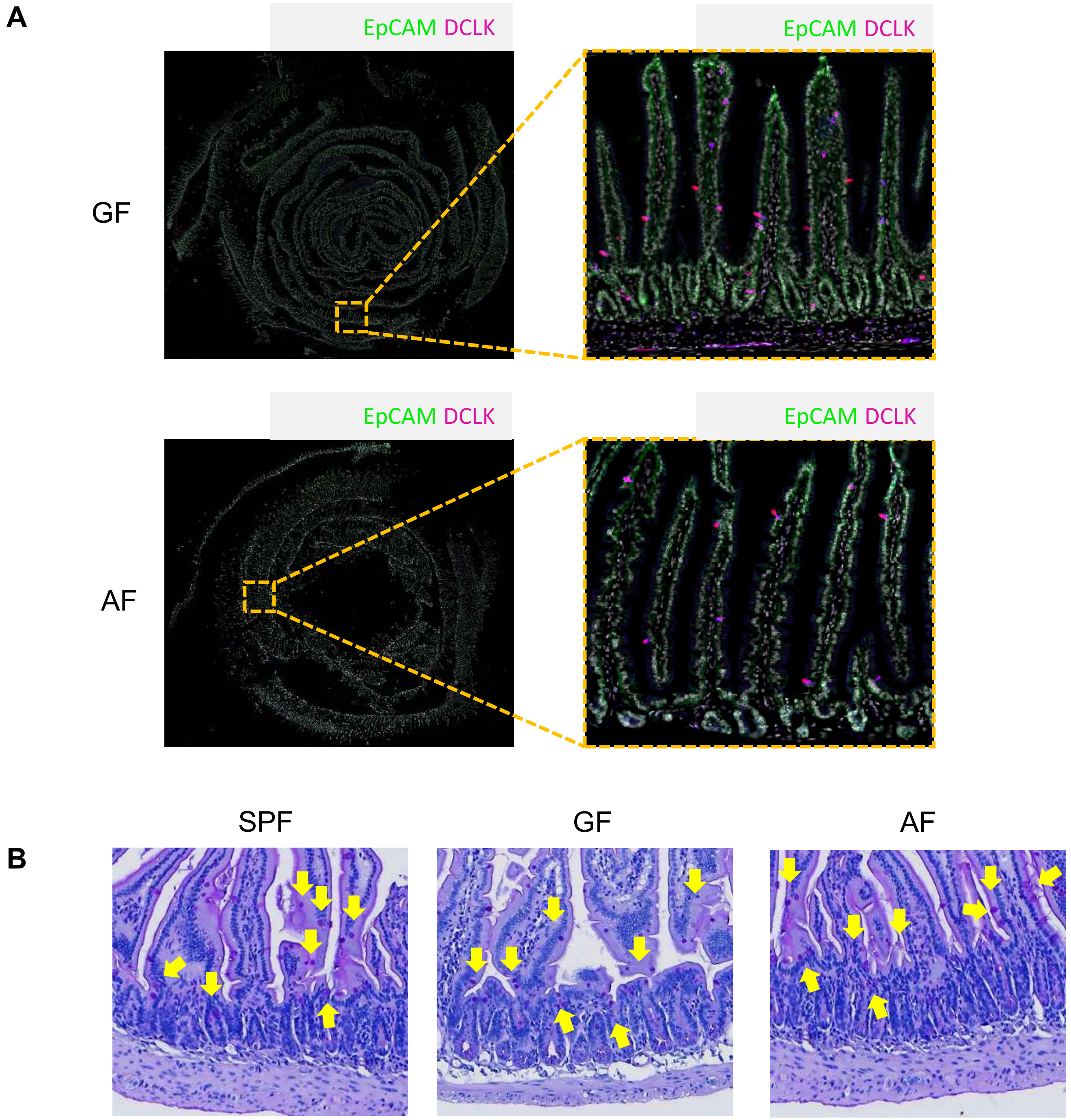

(A) Immunofluorescence analyses show DCLK+ tuft cells in small intestine of GF and AF mice. (B) PAS staining shows goblet cells in the small intestine of SPF, GF and AF mice. Results are from two independent experiments using 3 mice in each group.
