# Supplementary material for: Dietary antigens suppress the proliferation of type 2 innate lymphoid cells by restraining homeostatic IL-25 production
Source: Sci Rep. 2022 May 6;12:7443. doi: 10.1038/s41598-022-11466-4 (PMC9076687; doi:10.1038/s41598-022-11466-4)
Supplement: Supplementary file 3 — Supplementary Information 3. [file 41598_2022_11466_MOESM3_ESM.docx]

Supplementary Table 1. The table shows a complete list of significantly upregulated or downregulated genes in IECs of AF mice compared to GF and SPF mice.
